# Complete genome sequence of *Xanthomonas citri* pv. *mangiferaeindicae* GXBS06 isolated from the mango fruit in Guangxi, China

**DOI:** 10.1128/mra.01135-25

**Published:** 2025-12-12

**Authors:** Yi-Ming Li, Jie Peng, Yu Miao, Meijing Qin, Lixia Liao, Yinzi Tian, Shunan Wang, Yong-Qiang He, Wei Jiang

**Affiliations:** 1 State Key Laboratory for Conservation and Utilization of Subtropical Agro-Bioresource and College of Life Science and Technology, Guangxi University 12664, Nanning, China; 2 College of Agriculture, Guangxi University 12664, Nanning, Guangxi, China; University of Strathclyde, Glasgow, United Kingdom

**Keywords:** *Xanthomonas citri *pv. *mangiferaeindicae*, mango fruit, Illumina, PacBio

## Abstract

*Xanthomonas citri* pv. *mangiferaeindicae* (*Xcm*) GXBS06 was isolated from the mango cultivar “Guire 7” in Guangxi, China. Here, we report the complete genome sequence of strain *Xcm* GXBS06, which is composed of one chromosome and two plasmids.

## ANNOUNCEMENT

*Xanthomonas citri* pv. *mangiferaeindicae* (*Xcm*) is the causal agent of mango bacterial black spot (MBBS), which was first reported in South Africa in 1915, and the pathogen was identified as *Bacillus mangiferae* (1). In 2011, Pruvost et al. studied the IS1595 insertion sequence of the pathogen and concluded that it should be classified as *X. citri* (2). MBBS has now become the most severe bacterial disease affecting mangoes.

In this study, we report the genome of the *Xcm* GXBS06 strain from the mango cultivar “Guire 7” in the Chengbihu Mango Industry Demonstration Zone, Baise, Guangxi, China. A single diseased fruit was surface-sterilized with 75% (v/v) ethanol for 30 sec. One gram of tissue from a lesion was excised, homogenized in 10 mL of sterile water, and serially diluted. The dilutions were spread on nutrient agar (NA, 5 g/L polypeptone, 1 g/L yeast extract, 3 g/L beef extract, 1% (w/v) agar powder, pH = 7.0) plates and incubated at 28°C for 48 h. Single colonies were restreaked onto fresh NA plates and incubated at 28°C for 48 h for purification. After the isolate was cultured aerobically in nutrient broth (5 g/L polypeptone, 1 g/L yeast extract, 3 g/L beef extract, pH = 7.0) medium at 28°C with shaking at 200 rpm for 12 h, genomic DNA was extracted and purified using a QIAGEN Genomic-tip 20/G kit (Qiagen). For Illumina sequencing, a library was constructed using a VAHTS Universal Plus DNA Library Prep Kit for Illumina (Vazyme) and sequenced on an Illumina NovaSeq 6000 platform. To generate PacBio HiFi data, a separate isolation of genomic DNA was sheared using g-TUBEs (Covaris). A SMRTbell library with an average fragment size of 20 kb was constructed using a SMRTbell Express Template Prep Kit 3.0 (PacBio) without size selection and sequenced on the PacBio Sequel II system. All sequencing services were provided by Biomarker Technologies Co., Ltd. (Beijing). Raw reads were evaluated by FastQC v0.12.0 (3) and filtered with Trimmomatic v0.33 (4) (LEADING:3 TRAILING:3 SLIDINGWINDOW:50:20 MINLEN:100) for the Illumina reads and Filtlong v0.2.1 (min_length:2000, https://github.com/rrwick/Filtlong) for the PacBio reads. The filtered subreads were assembled by Canu v1.5 (5), corrected with the trimmed Illumina reads using Pilon v1.22 (6), and circularized with Circlator v1.5.5 (7) starting from the dnaA gene. Three circular contigs were assembled, and the two smaller replicons were identified as plasmids using Plasmer v0.1 (8). The genome completeness and contamination were assessed using CheckM v1.2.3 (9), yielding a completeness of 99.52% and a contamination of 0.12%, based on the *Xanthomonas* genus-level taxonomic marker set. The genome was annotated through the NCBI Prokaryotic Genome Annotation Pipeline v6.9 (10). The findings from the sequencing and annotation processes are summarized in Table 1.

**TABLE 1:** Genome characteristics of *Xcm*

Characteristic	Value		
Sequencing and assembly features	PacBio	Illumina	
No. of reads	238,834	10,517,836	
Total no. of bases (bp)	2,134,922,205	1,572,879,140	
Reads N50/read length (bp)	9,865	2 × 150	
Average genome coverage (×)	396.68	291.20	
Genome features	Chromosome	Plasmid1	Plasmid2
Size (bp)	5,277,303	83,456	40,491
GC content	64.70%	61.01%	61.45%
Number of non-coding RNAs	38	0	0
Number of tRNAs	52	0	0
Number of rRNAs	6	0	0
Total number of CDSs	4,469	83	51
CDSs with assigned function	4,193	68	48
CDSs assigned to COGs	3,648	0	0

Average nucleotide identity analysis, performed using the JSpeciesWS server (11), revealed a high degree of similarity between the isolate and the *Xcm* strain GXG07 (NCBI accession CP073209) at 99.92% (12), surpassing the standard 95% threshold for taxonomic classification of newly sequenced genomes. Default parameters were used for all software tools unless otherwise specified.

## Data Availability

The complete genomic sequence of the *Xcm* GXBS06 strain has been deposited in GenBank under accession numbers CP174366 (chromosome), CP174367 (plasmid 1), and CP174368 (plasmid 2). The raw sequencing data have been deposited in the NCBI Sequence Read Archive (SRA) under the accession numbers SRR33320586 (Illumina) and SRR33320587 (PacBio).

## References

[1] M. Doidge E. 1915. A bacterial disease of the mango, Bacillus mangiferae n.sp. Annals of Applied Biology 2:1–45. doi:10.1111/j.1744-7348.1915.tb05424.x

[2] Pruvost O, Vernière C, Vital K, Guérin F, Jouen E, Chiroleu F, Ah-You N, Gagnevin L. 2011. Insertion sequence- and tandem repeat-based genotyping techniques for Xanthomonas citri pv. mangiferaeindicae. Phytopathology 101:887–893. doi:10.1094/PHYTO-11-10-0304 21323466

[3] Andrews S. 2010. FastQC: a quality control tool for high throughput sequence data. Available from: http://www.bioinformatics.babraham.ac.uk/projects/fastqc

[4] Bolger AM, Lohse M, Usadel B. 2014. Trimmomatic: a flexible trimmer for Illumina sequence data. Bioinformatics 30:2114–2120. doi:10.1093/bioinformatics/btu170 24695404 PMC4103590

[5] Koren S, Walenz BP, Berlin K, Miller JR, Bergman NH, Phillippy AM. 2017. Canu: scalable and accurate long-read assembly via adaptive k-mer weighting and repeat separation. Genome Res 27:722–736. doi:10.1101/gr.215087.116 28298431 PMC5411767

[6] Walker BJ, Abeel T, Shea T, Priest M, Abouelliel A, Sakthikumar S, Cuomo CA, Zeng Q, Wortman J, Young SK, Earl AM. 2014. Pilon: an integrated tool for comprehensive microbial variant detection and genome assembly improvement. PLoS One 9:e112963. doi:10.1371/journal.pone.0112963 25409509 PMC4237348

[7] Hunt M, Silva ND, Otto TD, Parkhill J, Keane JA, Harris SR. 2015. Circlator: automated circularization of genome assemblies using long sequencing reads. Genome Biol 16:294. doi:10.1186/s13059-015-0849-0 26714481 PMC4699355

[8] Zhu Q, Gao S, Xiao B, He Z, Hu S. 2023. Plasmer: an accurate and sensitive bacterial plasmid prediction tool based on machine learning of shared k-mers and genomic features. Microbiol Spectr 11:e0464522. doi:10.1128/spectrum.04645-22 37191574 PMC10269668

[9] Parks DH, Imelfort M, Skennerton CT, Hugenholtz P, Tyson GW. 2015. CheckM: assessing the quality of microbial genomes recovered from isolates, single cells, and metagenomes. Genome Res 25:1043–1055. doi:10.1101/gr.186072.114 25977477 PMC4484387

[10] Tatusova T, DiCuccio M, Badretdin A, Chetvernin V, Nawrocki EP, Zaslavsky L, Lomsadze A, Pruitt KD, Borodovsky M, Ostell J. 2016. NCBI prokaryotic genome annotation pipeline. Nucleic Acids Res 44:6614–6624. doi:10.1093/nar/gkw569 27342282 PMC5001611

[11] Richter M, Rosselló-Móra R, Oliver Glöckner F, Peplies J. 2016. JSpeciesWS: a web server for prokaryotic species circumscription based on pairwise genome comparison. Bioinformatics 32:929–931. doi:10.1093/bioinformatics/btv681 26576653 PMC5939971

[12] Bie F, Li Y, Liu Z, Qin M, Li S, Dan X, Huang S, He YQ, Jiang W. 2022. High-quality genome resource of mango bacterial black spot pathogen Xanthomonas citri pv. mangiferaeindicae GXG07 isolated from Guangxi, China. Plant Dis 106:1027–1030. doi:10.1094/PDIS-08-21-1714-A 34633234

